# Advancing knowledge on the biogeography of arbuscular mycorrhizal fungi to support Sustainable Development Goal 15: Life on Land

**DOI:** 10.1093/femsle/fnaf055

**Published:** 2025-06-19

**Authors:** Justin D Stewart, Adriana Corrales, Cátia Canteiro, Clara Qin, Manju M Gupta, Burenjargal Otgonsuren, Clara P Peña-Venegas, Michael E Van Nuland, Petr Kohout, Tomáš Větrovský, Vasilis Kokkoris, Bethan F Manley

**Affiliations:** Society for the Protection of Underground Networks (SPUN), 3500 South DuPont Highway, Dover, DE 19901, United States; Amsterdam Institute for Life and Environment (A-LIFE), Section Ecology and Evolution, Vrije Universiteit Amsterdam, Amsterm, HZ 1081, the Netherlands; Society for the Protection of Underground Networks (SPUN), 3500 South DuPont Highway, Dover, DE 19901, United States; Society for the Protection of Underground Networks (SPUN), 3500 South DuPont Highway, Dover, DE 19901, United States; Fungal Conservation Committee, IUCN Species Survival Commission, Rue Mauverney 28, Gland 1196, Switzerland; Society for the Protection of Underground Networks (SPUN), 3500 South DuPont Highway, Dover, DE 19901, United States; Department of Biology, Sri Aurobindo College, University of Delhi, Delhi 110017, India; School of Earth and Sustainability, Department of Biological Sciences, Northern Arizona University, Flagstaff, AZ 86011, United States; Department of Ecology, School of Agroecology, Mongolian University of Life Sciences, Khan-Uul District, Zaisan, Ulaanbaatar 17024, Mongolia; Department of Forest Protection and Wildlife Management, Faculty of Forestry and Wood Technology, Mendel University in Brno, Zemědělská 3, Brno 61300, Czechia; Instituto Amazónico de Investigaciones Científicas SINCHI, Avenida Vasquez Cobo Between Calle 15, Leticia 910001, Colombia; Society for the Protection of Underground Networks (SPUN), 3500 South DuPont Highway, Dover, DE 19901, United States; Institute of Microbiology of the Czech Academy of Sciences, Vídeňská 1083, Prague 142 20, Czechia; Faculty of Science, Charles University in Prague, Albertov 6, Prague 128 00, Czechia; Institute of Microbiology of the Czech Academy of Sciences, Vídeňská 1083, Prague 142 20, Czechia; Amsterdam Institute for Life and Environment (A-LIFE), Section Ecology and Evolution, Vrije Universiteit Amsterdam, Amsterm, HZ 1081, the Netherlands; Society for the Protection of Underground Networks (SPUN), 3500 South DuPont Highway, Dover, DE 19901, United States

**Keywords:** arbuscular mycorrhizal fungi, biodiversity, Sustainable Development Goals, Life on Land, ecoregion, conservation

## Abstract

Arbuscular mycorrhizal (AM) fungi are fundamental to planetary health, enhancing plant nutrient uptake, stabilizing soils, and supporting biodiversity. Due to their prevalence and ecological importance, AM fungi are critical to achieving the environmental targets within the United Nations (UN) Sustainability Development Goals (SDGs) framework, including SDG 15: Life on Land. Despite these fungi engaging in the most widespread and ancient plant–microbe symbiosis, many fundamental aspects of the biogeography of AM fungi remain poorly resolved. This limits our ability to understand and document these fungal species’ contributions to preserving terrestrial life on Earth. Using the largest global dataset of AM fungal eDNA sequences, we highlight that > 70% of ecoregions have no available data generated from soil using AM fungal specific metabarcoding. Drawing attention to these severe data gaps can optimize future sampling efforts in key habitats. Filling these gaps and developing a more complete picture on the biogeographic distributions of AM fungal species will help to clarify their contributions to environmental targets.

## Teaser

Available arbuscular mycorrhizal fungal metabarcoding data are used to identify gaps and biases in global sampling, and their relevance to achieving Sustainable Development Goal 15 is discussed.

## Introduction

The United Nations’ (UN) Sustainable Development Goals (SDGs) are a global framework designed to drive progress toward a more sustainable and equitable future. Adopted by 191 UN member states, the SDGs consist of 17 goals addressing urgent global challenges, from poverty and hunger to climate action and biodiversity conservation (United Nations 2025). Among these, SDG 15: Life on Land focuses on protecting, restoring, and promoting the sustainable use of terrestrial ecosystems. SDG 15 includes key targets such as ensuring the conservation of terrestrial and freshwater ecosystems (15.1), restoring degraded ecosystems (15.2), combating desertification and restoring degraded land and soil (15.3), and halting biodiversity loss (15.5)—all of which require a deeper understanding of the biological systems that maintain ecosystem health.

Current biodiversity policies and conservation strategies disproportionately focus on well-studied organisms such as birds and mammals, while microbial communities—key players in soil health, nutrient cycling, and ecosystem stability—are rarely considered (Troudet et al. 2017, Guerra et al. 2020, Hughes et al. 2021). Even among microbial taxa, discussions tend to center on bacteria, with fungi receiving minimal attention (Timmis et al. 2017, Akinsemolu 2018, George and Ray 2023, Crowther et al. 2024, Nature Microbiology Editorial Team 2025). This oversight is especially stark for arbuscular mycorrhizal (AM) fungi, a globally distributed group of microscopic soil fungi that play an integral role in plant productivity and ecosystem functioning yet are not visible aboveground (Wilson et al. 2009, Wagg et al. 2011, Brundrett and Tedersoo 2018, Soudzilovskaia et al. 2019, Steidinger et al. 2019, Braghiere et al. 2021, Shen et al. 2023).

AM fungi form arguably the most widespread plant–microbe symbioses on Earth, forming mutualistic associations with over 70% of terrestrial plant species (Brundrett and Tedersoo 2018). AM fungi are composed of tubular-like structures called hyphae, which extend into the soil, accessing nutrients such as phosphorus and nitrogen that would otherwise be limiting to plants. These hyphae colonize plant roots, where nutrients are transported back to the host plant in exchange for carbon, with AM fungi supplying up to 80% of a plant’s phosphorus and 20% of its nitrogen needs (Marschner and Dell 1994, Leigh et al. 2009, Barrett et al. 2011, Thirkell et al. 2016, Andrino et al. 2021, Etesami et al. 2021). This symbiosis is responsible for a massive global carbon flux, with an estimated 1 billion tons of carbon allocated annually from plants to AM fungal hyphae (Hawkins et al. 2023). AM fungi likely played a key role in the development of life on land by aiding plants in the transition from aquatic to terrestrial environments over 450 million years ago (Redecker et al. 2000, Field et al. 2015). The local biodiversity of AM fungi in soils has been connected to the biodiversity and productivity of plant communities aboveground (Van Der Heijden et al. 1998, Wagg et al. 2011, 2014); although these linked diversity patterns become less clear at global scales (Toussaint et al. 2020). Due to their long coevolution with terrestrial plants, AM fungi have become a keystone component in planetary-scale nutrient cycling, influencing soil carbon storage, plant productivity, and ecosystem resilience, making AM fungi critical to multiple SDG 15 targets (Leake and Read 2017, Delavaux et al. 2019, Braghiere et al. 2022, Hawkins et al. 2023).

Scientists face unique challenges when studying AM fungi that hinders quick integration of this group into conservation and sustainability assessments (Hansen et al. 2013, Kays et al. 2015). Unlike forests, certain vertebrate populations and many macrofungi (e.g. Agaricomycetes), AM fungi cannot be directly observed or monitored via remote imagery. As obligate symbionts that rely on plant roots for survival, they are difficult to isolate and culture in the lab (Smith and Read 2008). Their belowground nature and lack of fruiting bodies make them less visible to researchers or community scientists for recording natural observations.

Standard microbial sequencing methods often fail to capture their full diversity due to primer biases (Tedersoo et al. 2015, 2018, Lutz et al. 2025). Studies that use eDNA to survey soils often rely on “general” fungal primers (rather than AM fungi-specific primers) and may miss species of AM fungi by amplifying and sequencing more abundant soil fungi (Tedersoo et al. 2014, 2015, Lekberg et al. 2018). While advances in eDNA sequencing and curated fungal databases provide promising tools for AM fungal detection, historical biases in primer and marker choice in soil microbiome assessments continue to limit our understanding of their true global distribution (Delavaux et al. 2021, Větrovský et al. 2023, Tedersoo et al. 2024, Corradi et al. 2025). Even when using traditional morphological assessment of spores, a time-consuming method that requires soil slurry filtration and detailed microscopy analysis of single spores, misidentifications and biases are common (Bentivenga et al. 1997). Though several studies have used spore morphology for the purposes of AM fungal biogeographic analyses, we focus on eDNA due to its high-throughput nature, the existence of large soil sequencing databases, and potential for data uniformity across studies (Pringle and Bever 2002, Ananthakrishnan et al. 2004, Albaqami et al. 2018, Stürmer et al. 2018, Moura et al. 2022). Still, many basic aspects of AM fungal biogeography are unknown. However, the extent to which AM fungi contribute to SDG targets may ultimately depend on the spatial distribution of their biodiversity, endemism, and individual species ranges.

Addressing knowledge gaps in the field of AM fungal biodiversity and biogeography is essential for integrating belowground biodiversity into conservation efforts, ensuring that AM fungi are recognized in their contributions to achieving global sustainability goals. In this review, we aim to: (i) discuss the contributions of AM fungi to SDG 15 “Life on Land,” (ii) use available soil eDNA data to highlight geographic regions, where AM fungi remain underrepresented in global sampling of Earth’s soils, and (iii) identify challenges in translating biogeographical data on AM fungal distributions into tangible conservation protections.

## AM fungi are a crucial component for Life on Land

**Figure 1.**
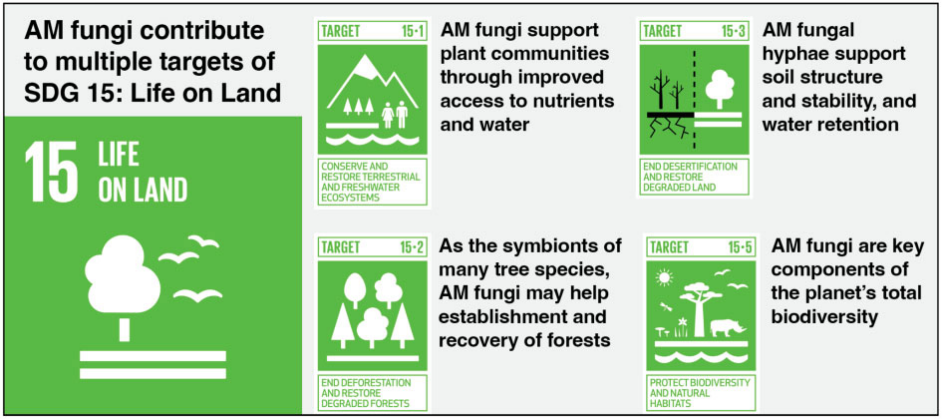
The role of AM fungi in supporting Sustainable Development Goal 15: Life on Land: AM fungi contribute to multiple SDG 15 targets, including Target 15.1 conserving and restoring terrestrial and freshwater ecosystems, Target 15.2 ending deforestation and restoring degraded forests, Target 15.3 ending desertification and restoring degraded lands, and Target 15.5 protecting biodiversity and natural habitats.

Central to the SDG 15: Life on Land is the recognition that Earth’s biodiversity is an immeasurable asset to humanity (Arora and Mishra 2024). Imbalances in terrestrial ecosystems caused by the biodiversity crisis could lead to tipping points that irreparably damage ecosystems, with disastrous consequences for both natural and human systems (World Wide Fund for Nature Living Planet Report 2024). As symbionts of a majority of terrestrial plant species, AM fungi are not only key components of biodiversity but also support entire ecosystems (Van Der Heijden et al. 2008, Wagg et al. 2011). This section explores how AM fungi may contribute to SDG 15 by supporting targets related to the conservation of terrestrial ecosystems, including targets 15.1 (conservation of terrestrial ecosystems and preventing land degradation), 15.2 (forest restoration), 15.3 (combating desertification and restoring degraded land), and 15.5 (halting biodiversity loss) by influencing key ecosystem functions that sustain plant life, soil health, and nutrient cycling (Fig. 1).

AM fungi are overlooked in their potential contributions to the conservation of terrestrial ecosystems (SDG target 15.1). In forests, AM fungi can enhance seedling establishment, promote tree growth, and improve nutrient uptake (Janos 1980, Wall et al. 2020, Martius et al. 2024). They may also be a key component for restoration of degraded ecosystems (Bever et al. 2001). The ability of AM fungi to support tree establishment aligns with reforestation efforts, particularly in degraded or nutrient-depleted landscapes (SDG 15.2). AM fungi may also help protect rare and endangered plants from extinction (Bothe et al. 2010). These contributions may be especially important in tropical regions, where deforestation rates are high, and many AM host plants are present (Soudzilovskaia et al. 2019, Pendrill et al. 2022). There is an ongoing discussion on the use of mycorrhizal inoculants or the introduction of plants colonized with AM fungi to facilitate mycorrhizal symbioses in new seedlings as a method of improving restoration success (Hart et al. 2018). These methods could increase seedling establishment rates and enhance ecosystem diversity if done correctly (Perring et al. 2015, Wall et al. 2020, Martius et al. 2024). Restoration initiatives could benefit from using native AM fungal species as inoculants for a particular ecosystem, as native AM fungal species may provide increased benefits to native plant species (Koziol et al. 2022a, Ouahmane et al. 2007, Rúa et al. 2016). However, widely available commercial inoculants that consist of a small number of easily propagated species present risks of spreading nonnative AM fungal species (Basiru and Hijri 2022, Koziol et al. 2024). The use of such inoculants directly threatens SDG 15.8, which aims to prevent the introduction of invasive species. While further research is needed into the efficacy of AM fungal inoculum, there is growing evidence that inoculum consisting of native AM fungal species have positive impacts on local plant growth, which may be negatively impacted when commercial, nonnative inoculum is used (Koziol et al. 2022b, 2023). Detailed knowledge on the biogeographical distribution of AM fungal species at different spatial scales would help establish baselines for monitoring the potential return of local key species of AM fungi to landscapes undergoing restoration (Wall et al. 2020). This knowledge would also enable more thoughtful production and application of mycorrhizal inoculants for agricultural systems using native species (Kuila and Ghosh 2022).

The extensive hyphal networks of AM fungi physically bind soil particles together, reducing wind erosion, a primary driver of desertification (Burri et al. 2013). In ecosystems where water is scarce and nutrient mobility in soils is low, AM fungi extend hyphae beyond the root zone, increasing plant access to water and nutrients (CUI and NOBEL 1992, Kakouridis et al. 2022). This role is particularly important for stabilizing dryland ecosystems, where plants are challenged by frequent periods of drought and low nutrient mobility in desert soils (Allen 2007). In many grasslands, AM fungi are thought to be one of the most important pathways for plant nitrogen uptake (Braghiere et al. 2022). This nutrient uptake is likely important to promoting plant growth in grassland ecosystems, many of which are at risk of desertification (Bardgett et al. 2021). AM fungi should therefore be considered in efforts to prevent further land degradation and combat desertification (SDG target 15.3). Still, we do not have a consensus on what interventions best support AM fungi toward this SDG target. Research is needed to identify when and where interventions, such as inoculations, are successful in these ecosystems.

AM fungi should also be factored into concerns over biodiversity loss, as important constituents of total biodiversity (SDG target 15.5), and should be considered as part of a solution to this issue. Aboveground, research has shown that AM fungi often increase plant biomass and are likely required by many plant species for survival, with potential positive impacts on the many organisms that rely on vegetation, including invertebrates (Hoeksema et al. 2010). AM fungal diversity in soils is associated with plant diversity aboveground, meaning that soils with more diverse AM fungal communities tend to support greater plant species richness (Van Der Heijden et al. 1998, 2008, Wagg et al. 2011). However, the relationship between AM fungal diversity and host plant benefits is complex and can change over space and time, and more research is needed to understand this context dependency (Kokkoris et al. 2020). AM fungi are a biological interface for the flow of plant-derived carbon from aboveground to belowground ecosystems. AM fungi allocate photosynthetically fixed carbon to their hyphal networks which in turn support a diverse community of bacteria, other fungi, and soil microfauna such as springtails and earthworms (Gormsen et al. 2004, Siddiky et al. 2012, See et al. 2022). In some soils, such as in grasslands, hyphae can reach up to ∼100 m in a single cm^3^ of soil, creating a large physical habitat for soil life (Miller et al. 1995). However, mycorrhizal fungi are underutilized in habitat management and restoration and largely overlooked in land management plans (Markovchick et al. 2023).

## Gaps in AM fungal biogeography data

An accurate and up-to-date global inventory of AM fungal biodiversity is necessary to assess their ecological roles at scale. There are several challenges associated with AM fungal taxonomy and identification, and there are very few taxonomy specialists working on this group, which slows down the rate of species discovery and description. Increasing the number of AM fungi described, which currently sits at around 360 species (Schüßler and Walker 2024), will require expanding local expertise, supporting culture collections, and robust eDNA analyses (Hyde and Noorabadi 2024, Kolaříková et al. 2021, Stürmer et al. 2021, Niezgoda et al. 2024, Tedersoo et al. 2024, Corradi et al. 2025). Until the adoption of eDNA metabarcoding (Davison et al. 2015), the biogeographical distributions of fungi were understood through the collections of physical specimens such as mushrooms, ignoring more cryptic groups such as AM fungi. Broadening our available biogeographic data using metabarcoding methods optimized to identify AM fungi and creating better species definitions will likely unlock the full biodiversity patterns within this group. Prior efforts may be overemphasizing the level of cosmopolitan distribution (Davison et al. 2015, Bruns and Taylor 2016).

Global biodiversity maps are essential tools for understanding regional ecology and guiding conservation efforts. Area-based conservation strategies use data on biodiversity hotspots and protected areas (SDGs targets 15.1 and 15.4), and these areas are delimited based on data from individual species, their distributions and extinction risks (Darbyshire et al. 2017, Maxwell et al. 2020). Comparing fungal biodiversity in protected versus unprotected areas (e.g. using counter-factual analysis methods) could provide critical insights into how habitat fragmentation, deforestation, and agricultural expansion alter underground biodiversity (Naughton-Treves et al. 2005, Gray et al. 2016). Such analyses are vital for identifying regions, where AM fungal diversity is declining and implementing conservation actions that enhance AM fungal contributions to SDG targets. Organizations including the World Wildlife Fund (WWF) use area-based conservation action plans, which rely on ecoregions as fundamental units for biodiversity protection and conservation (Morrison et al. 2009). These action plans assess species’ geographic distributions, linking them to environmental gradients and potential threats to develop targeted conservation strategies. However, the uneven distribution of sampled soils on Earth presents a major challenge to completing this first step of comprehensively mapping the biogeographical distributions of AM fungi, leaving significant gaps in our ability to integrate belowground biodiversity into conservation assessments, planning and action (Reddy and Dávalos 2003, Oliveira et al. 2016, Hughes et al. 2021).

A major challenge for biodiversity mapping efforts is to pinpoint where new samples should be collected, particularly as resources for biodiversity mapping can be limited and efforts must be strategic (Hortal and Lobo 2005, Antonelli et al. 2024). This sampling optimization relies on defining a meaningful spatial unit to differentiate unique communities and patterns across environmental gradients. One option is to categorize global biodiversity into so-called ecoregions, designated according to their unique terrestrial biodiversity, environmental conditions, and known drivers of speciation (Olson et al. 2001, Dinerstein et al. 2017). For animals and plants, ecoregions help to define species accumulation patterns. Fungi show weaker, but still significant correlations to ecoregion borders, with the weaker correlation likely due to poor species definition or identification and low sample sizes (Smith et al. 2018).

To assess the extent to which Earth’s 848 distinct ecoregions have been sampled specifically for AM fungi, we summarized the spatial distribution of soil samples from GlobalAMFungi database. This is the most comprehensive public dataset of AM fungal communities captured using taxon specific primers that amplify the most common marker regions used to identify AM fungi (Větrovský et al. 2023). Although root samples are available in this database, soil samples were used for this analysis as bulk soil samples are often used in large-scale mapping studies, and roots host communities of AM fungi that are dominated by the family Glomeraceae, while soils host higher diversity of AM fungal families (Tedersoo et al. 2014, Mikryukov et al. 2023, Van Nuland 2025). Likewise, the small subunit (SSU) region of ribosomal RNA (rRNA) was chosen for this analysis as this is the marker with the highest representation in the GlobalAMFungi database, with 3234 soil samples sequenced using AM fungal-specific primers that amplify the SSU marker. Although AM fungi can be identified using the more common universal fungal primer sets that target the internal transcribed spacer (ITS) region, studies have identified that SSU and large subunit regions are more effective at taxonomic placement due to high variability in the ITS region in AM fungi (Stockinger et al. 2010). A future step for producing more robust AM fungal biogeography data could involve generating longer metabarcoding sequence data that covers more than one of these rRNA regions (Lutz et al. 2025).

Briefly, we used the GlobalFungi database of geolocated soil samples sequenced using primers targeting the SSU rRNA region and counted the samples collected from each RESOLVE ecoregion using R (v. 4.43) (Olson et al. 2001, Gorelick et al. 2017, Větrovský et al. 2023). Data and code for this analysis are available on Github (https://github.com/SocietyProtectionUndergroundNetworks/UnderexploredEcosystems).

**Figure 2.**
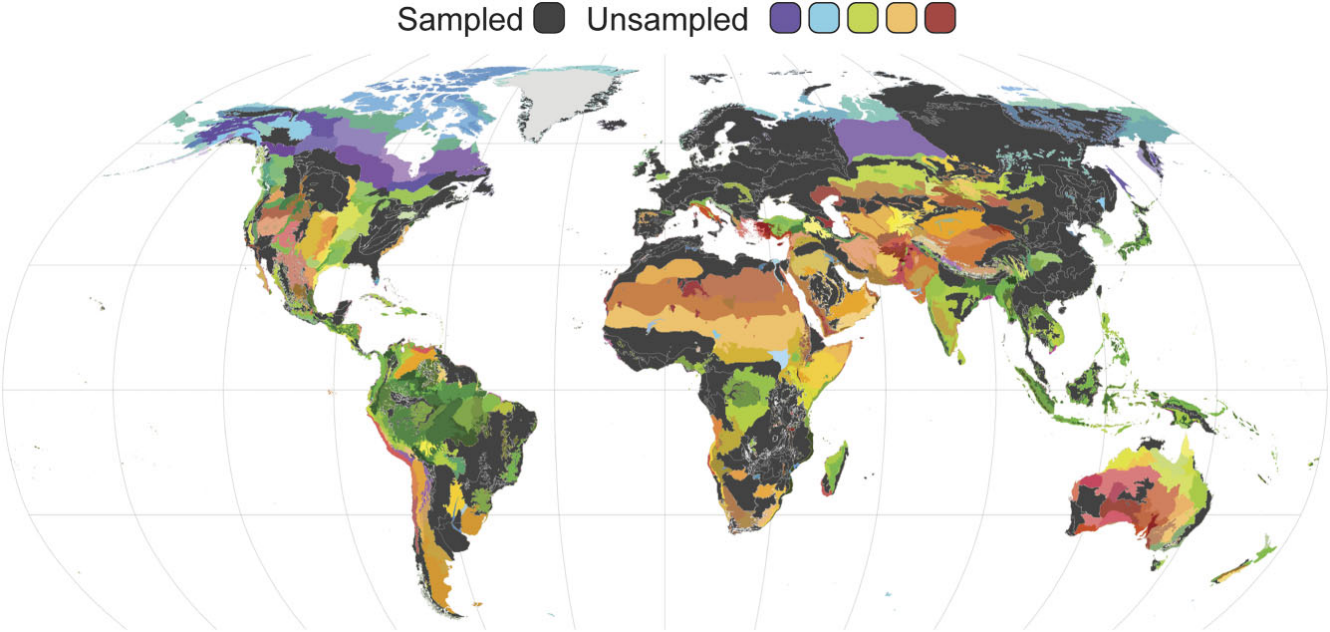
Over 70% of ecoregions are undersampled for AM fungi: Map of the 848 RESOLVE ecoregions colored by sampling for AM fungi. Sampling data are from the GlobalAMFungi database of SSU sequences (Větrovský et al. 2023), the most widely used AM-fungal specific marker. Of the Earth’s 848 ecoregions, only 230 have been sampled for AM fungi, leaving 73% of all ecoregions unsampled (color scale). Colors in this map correspond to those in the ecoregions app where information on local flora, fauna, and climate can be accessed. information on ecoregions and colors can be accessed at https://ecoregions.appspot.com/. For ecoregions that have been sampled, an average ∼4 samples have been collected, however this varies from 1 to 329 samples between different ecoregions. Individual ecoregions with sample counts can be found in supplemental data. Images were sourced from the authors, Wikimedia, and the RESOLVE database (RESOLVE 2025).

**Figure 3.**
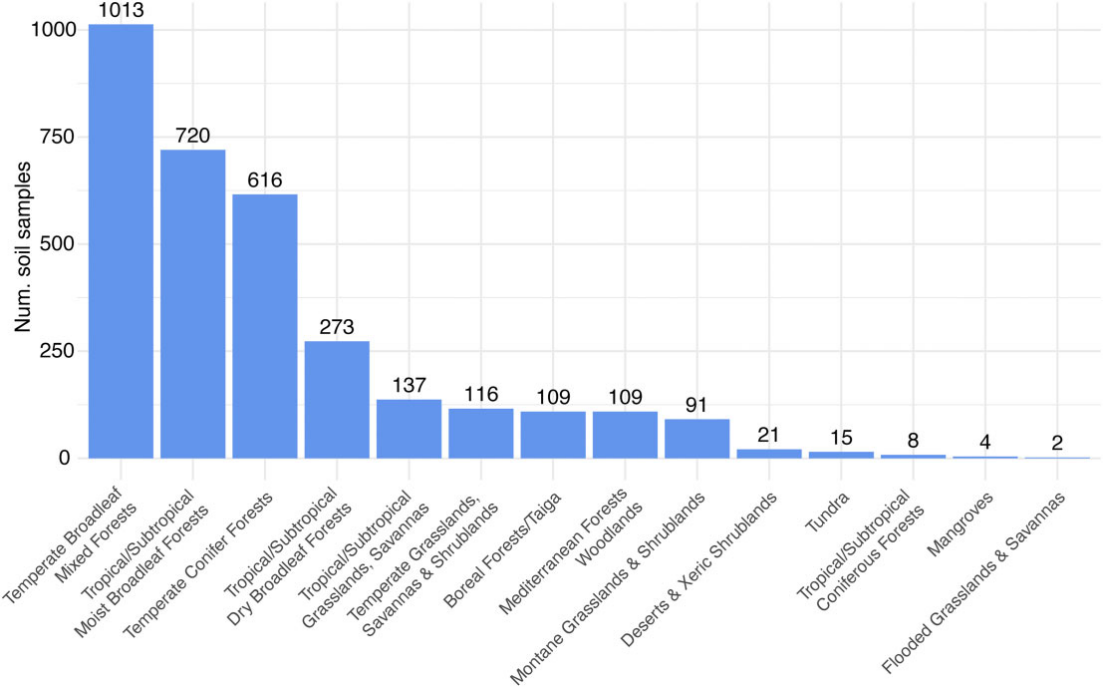
Biome biases in number of soil samples sequenced for AM fungi in the GlobalAMFungi database: Bars represent the total number of samples collected in a given biome, with the exact number of samples above each bar. Similar to sampling biases at the ecoregion scale, temperate and tropical forests represent some of the most sampled biomes with the highest number of sample counts, while drylands, mangroves, and flooded grasslands are some of the least sampled biomes. A list of sample counts per ecoregion and biome is available in Supplemental File 1.

**Figure 4.**
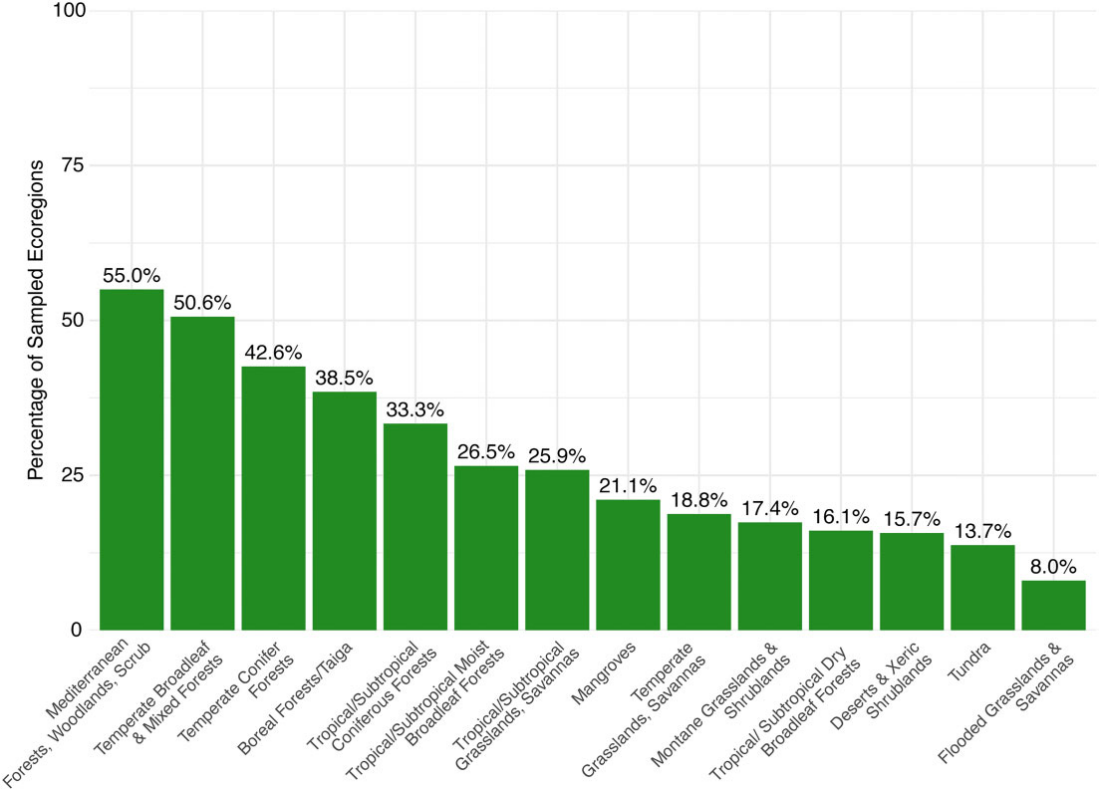
Percentage of ecoregions sampled varies between biomes: The proportion of sampled ecoregions varies across biomes, reflecting differences in sampling effort of data in the GlobalAMFungi database. Bars represent the percentage of ecoregions within each biome that contain at least one sample, with values displayed at the top of each bar. Sampling biases are evident, with Mediterranean and temperate ecosystems showing the highest coverage—over 50% of ecoregions sampled—while flooded grasslands and savannas remain the least sampled biome. These disparities highlight the uneven distribution of soil biodiversity data across global ecosystems.

We found that 73% (617 ecoregions) of the Earth’s ecoregions are completely unsampled for AM fungi in soils (Figs 2 and 3). Overall, most ecoregions could be considered undersampled (with <5 samples) and there was a significant geographical bias in sampling effort, also at the biome scale (Fig. 3). Over half of all ecoregions found in Mediterranean forests, woodlands, and scrublands biome have been sampled (55%) as well as in temperate broad leaf mixed forests biome (50.6%). In comparison, less than a quarter of ecoregions have been sampled in mangroves, temperate grasslands and savannas, tropical/subtropical broadleaf dry forests, desert and xeric shrublands, tundra soils, and flooded grasslands (Fig. 4). This uneven distribution and small number of soil samples sequenced specifically for AM fungi is unsurprising, as biases in soil biodiversity sampling have been highlighted previously (Guerra et al. 2020).

Notable ecoregions that are underrepresented in global datasets that target AM fungi include those within drylands, which can include deserts, steppe areas, and dry grasslands, yet these systems cover ∼45% of the Earth’s terrestrial surface (Olson et al. 2001, Dinerstein et al. 2017). Examples of these ecoregions are the Kazakh steppe, Chihuahuan Desert, and Sahelian Acacia Savanna. Given the low vegetation cover in these ecosystems there may be biases to sampling in these ecoregions. Many existing models of mycorrhizal fungal biogeographic distributions exclude desert regions or assign them high uncertainty estimates, limiting our ability to accurately model AM biodiversity across a significant portion of Earth’s landscapes (Mikryukov et al. 2023, Van Nuland 2025). Further, the satellite sensors used to measure key predictors of AM fungal biodiversity, such as climate and net primary productivity, are too coarse in resolution (e.g. 250 m^2^–1 km^2^) to capture the sparsely distributed vegetation where most dryland AM fungi are likely found. As advances in remote sensing improve our ability to map ecosystems at the resolution of individual plants, identifying regions where AM fungi are scarce or absent will be just as informative as pinpointing areas of high fungal diversity.

Urban ecosystems also tend to be undersampled habitats for soil biodiversity (Rega-Brodsky et al. 2022, Stewart et al. 2024, Verbeek et al. 2025) These areas cover ∼3% of terrestrial land while hosting over half of the human population (Zhou et al. 2015). By enhancing plant biomass production, improving stress tolerance, and facilitating nutrient cycling, AM fungi can help urban plants persist under the extreme conditions of cities including habitat fragmentation, heat, and pollution exposure (Gupta et al. 2018, Stewart et al. 2024, Verbeek et al. 2025). Cities may be among the easiest blind spots to fill in sampling gaps. With the majority of the human population living in cities, there are growing opportunities for researchers and even citizen scientists to collect AM fungal samples using novel microbial sampling techniques (Meyer et al. 2021, Silva-Flores et al. 2021, Stewart et al. 2021).

Advancements in machine learning have enabled researchers to account for these uneven sampling distributions, allowing for high-resolution predictive mapping of AM fungal biodiversity across Earth’s ecosystems (Mikryukov et al. 2023, Van Nuland 2025). These maps rely on geo-located samples of AM diversity, which are used to train models that interpolate and extrapolate diversity estimates into unsampled soils (Hoogen et al. 2021). While these advancements are promising, models remain subject to high uncertainty and extrapolation—particularly in remote regions far from where samples have been taken (Mikryukov et al. 2023, Van Nuland 2025). These models are most reliable in areas with sufficient training data, and predictions of AM fungal diversity in regions with poor sampling coverage become increasingly uncertain and prone to inaccuracies (Ploton et al. 2020, Meyer and Pebesma 2022, Milà et al. 2022). Critically, improving model reliability is not just a matter of increasing sample size, but of strategically collecting new samples to expand training data coverage across a broader range of habitats, allowing models to rely more on interpolation rather than extrapolation of biodiversity patterns (Meyer and Pebesma 2021, 2022).

Filling in data gaps for AM fungi may not always require new sampling. Many global databases primarily compile studies published in English, limiting the inclusion of non-English research and creating biases in sample distributions (Konno et al. 2020). Recent efforts to create inclusive data repositories and international working groups offer ways to integrate these overlooked datasets into broader research efforts (Silva-Flores et al. 2021, Tedersoo et al. 2021). In fact, advancements in translation technology and automated data extraction now make this integration more feasible than ever (Gougherty and Clipp 2024). Expanding biodiversity databases to include multilingual datasets would significantly improve our ability to map and understand global AM fungal diversity. Increased diversity in who carries out research into AM fungal biogeographical studies should also be an aim for improving mapping of these fungi. Fair and equitable north–south collaborations involving local AM fungal experts may facilitate sampling of undersampled ecoregions.

## Translating AM fungal data into action

Generating data on the biogeographical distributions and ecosystem contributions of species can lead to predictions about the vulnerability of organisms and ecosystems, where these predictions can inform mitigation or management actions (Chen et al. 2024). Such outcomes rely on accurately understanding biogeographical distributions, and there have been numerous examples within the fungal kingdom, where species were considered to be geographically widespread, until molecular data revealed that these were groups of multiple species, each with limited distributions (Niskanen et al. 2023). Without efforts to fill in biodiversity information gaps and understand the localized distributions of AM fungi, they will continue to be overlooked in policies aimed at protecting ecosystems and maintaining biodiversity under increasing environmental pressures. In addition to the SDGs, AM fungi are also key to achieving other multilateral environmental agreements, including the Kunming-Montreal Global Biodiversity Framework. However, fungi in general have been mostly absent from conservation policy and action (Cao et al. 2021, Gonçalves et al. 2021). Many of these agreements include targets related to stopping or reducing species extinction, and their progress indicators, as well as most conservation action on the ground, depend on understanding which species are at higher risk of extinction, how different threats impact them, and where.

Many challenges must be considered and overcome before AM fungal communities can be better understood and protected. Current efforts to integrate AM fungi into conservation planning remain severely limited. Based on recent global AM fungal richness maps, 95% of AM fungal biodiversity hotspots are located in soils outside protected areas, highlighting a significant gap in conservation strategies (Turrini and Giovannetti 2012, Van Nuland 2025). Without incorporating functional diversity, ecosystem roles, and spatial protection assessments, AM fungal conservation will remain overlooked despite their critical contributions to soil health, plant biodiversity, and ecosystem stability. Understanding which fungi are present in different locations will be essential as trait databases for AM fungal functions are developed (Chaudhary et al. 2025). Similarly, sampling AM fungal communities can be synergistic with measuring ecosystem functions, such as the density of hyphae or spores in soils, which can serve as proxies for soil aggregate stability and biomass (See et al. 2022).

The IUCN Red List of Threatened Species (hereafter Red List) is often used as the main indicator for biodiversity conservation action and policy, including for target 15.5 of the SDGs. Still, the Red List has significant gaps in species representation, with only 1300 fungal species listed, a very small proportion of the ∼155 000 scientifically described fungal species, and even less of the estimated total ∼2–3 million species on Earth (Niskanen et al. 2023, IUCN 2025b). Most of these red listed species are Basidiomycota or lichenized Ascomycota—no AM fungi are included (Mueller et al. 2022). This is in part a result of knowledge gaps, with more than 90% of the estimated total number of fungal species being taxonomically undescribed, including AM fungi (Schüßler and Walker 2024). A further obstacle relates to the Red List methodology, as its criteria can be applied to any taxa across the different kingdoms of life, except for microbes (IUCN Standards and Petitions Committee 2025a). This excludes all AM fungi and other key microscopic species that sustain biodiversity, species that are too small for us to see but too important for us to lose. Despite ongoing discussions on how to adapt the Red List to microbial life, no standard guidelines have been produced. As we monitor progress and come closer to the deadline for achieving SDGs and other global conservation targets, it is important to use adequate indicators that reflect the conservation status of all biodiversity, either by adapting the Red List guidelines or finding alternative indicators for AM fungi and other microbes.

Targeted efforts to sample unsampled soils are a way of laying the groundwork for AM fungi to eventually be included in different conservation programs such as the IUCN Red List and global biodiversity frameworks. As advancements in DNA sequencing, ecological modeling, and remote sensing continue to improve, these efforts should not only focus on addressing existing knowledge gaps but also take a forward-facing approach that incorporates emerging technologies. Expanding fungal biodiversity assessments will improve conservation strategies for AM fungi while safeguarding their contributions to life on land. However, to fully integrate AM fungi into conservation efforts, we still need to identify how their biodiversity is distributed across soil ecosystems. Addressing these gaps will be essential for ensuring that AM fungi are protected alongside other key components of life on land. To fill these gaps and effectively achieve SDG goals that factor in all biodiversity, it is imperative to involve planning agencies, scientists, and local communities. This collaborative effort will be vital in ensuring the sustainable management and preservation of ecosystems.

## Supplementary Material

fnaf055_Supplemental_File
